# The association between cognition and gait in a representative sample of very old people – the influence of dementia and walking aid use

**DOI:** 10.1186/s12877-020-1433-3

**Published:** 2020-01-31

**Authors:** Jerry Öhlin, Anders Ahlgren, Robert Folkesson, Yngve Gustafson, Håkan Littbrand, Birgitta Olofsson, Annika Toots

**Affiliations:** 1 Department of Community Medicine and Rehabilitation, Geriatric Medicine, Umeå University, SE-90187 Umeå, Sweden; 2 Department of Nursing, Umeå University, SE-90187 Umeå, Sweden; 3 Department of Community Medicine and Rehabilitation, Physiotherapy, Umeå University, SE-90187 Umeå, Sweden

**Keywords:** Gait speed, Cognition, Walking aids, Dementia, Aged 80 and over

## Abstract

**Background:**

Cognition has been related with gait speed in older adults; however, studies involving the oldest age group, where many have mobility disability and cognitive impairment, are few. The aim was to investigate the association between global cognitive function and gait speed in a representative sample of very old people, and whether the association was affected by dementia, and walking aid use.

**Method:**

This cross-sectional study included 1317 participants, mean age 89.4 years, and 68% women, from the Umeå85+/Gerontological Regional Database. Self-paced gait speed was measured over 2.4 m, with or without walking aids, and global cognitive function with the Mini-Mental State Examination (MMSE). The association between cognition and gait speed was analyzed using multiple linear regression and stratified according to dementia. The influence of missing gait speed values was explored using multiple imputation. An interaction analysis was performed to investigate the influence of walking aid use.

**Results:**

In comprehensively adjusted analyses, MMSE associated with gait speed (unstandardized β (β) 0.011 m/s, 95% Confidence Interval [CI] = 0.009, 0.013, *p* < 0.001) in the total sample. No association was found in people with dementia (β 0.003 m/s, 95%CI = 0.000, 0.006, *p* = 0.058), until missing gait speed values were compensated for by multiple imputation (β 0.007 m/s, 95% [CI] = 0.002, 0.011, *p* = 0.002). In interaction analysis the use of walking aids attenuated the association between cognition and gait speed (β − 0.019 m/s, 95%CI = − 0.024, − 0.013, *p* < 0.001).

**Conclusion:**

Global cognitive function appears to associate with gait speed in very old people. However, in people with dementia selection bias was indicated since unless missing gait speed values were accounted for no association was observed. Walking aid use attenuated cognitive load, which may not apply to walking in daily activities, and requires further investigation.

## Introduction

The number and proportion of older people are increasing, with the fastest growing age group being people aged 80 years or older. By 2050, this age group will comprise 20% of the older population, having increased threefold [1]. In higher age the prevalence of dementia and other age-related diseases increase. Gait speed deteriorates with age, and a slower gait speed is associated with numerous negative health consequences, e.g. increased dependence in activities of daily living (ADL) [2], falls [2], dementia [3], and mortality [4]. Subsequently, much research has focused on investigating factors associated with gait speed to support development of effective prevention and rehabilitation [5].

Gait is a complex task dependent on the interplay between multiple systems including cognition [4]. Previous studies have found a positive association between gait and cognition [6, 7] with a slower gait speed associated with a decline in cognitive function [8]. However, amongst very old people, including those with dementia, and those living in nursing homes, evidence is limited because studies are few and small [7], despite gait and cognitive dysfunction being more common [9] and severe [10, 11] in these groups. Furthermore, the use of walking aids is more common among people of higher age [12]. Walking aids improve gait by alleviating pain or compensating for deficits in balance [13] and may thus lower the cognitive challenge of the motor task and increase gait speed [14]. Conversely, during more challenging motor tasks requiring greater maneuvering of the walking aid, it may increase the cognitive challenge, thus lowering gait speed [15]. In addition, the occurrence of multimorbidity (having at least two chronic diseases) increases with age [16]. Many medical conditions are associated with both gait and cognition, and it therefore seems important to investigate if the association remains when potential confounders are adjusted for. Finally, a relevant proportion of the older population have impaired mobility [17]. This may lead to difficulties performing gait speed tests according to protocol resulting in missing values, and compromising the generalizability of results.

The aim of this study was to investigate the association between gait speed and global cognitive function in a representative sample of very old people, including people with dementia, and people living in nursing homes, and whether using walking aids during the gait speed test affects the association.

## Method

**Fig. 1:**
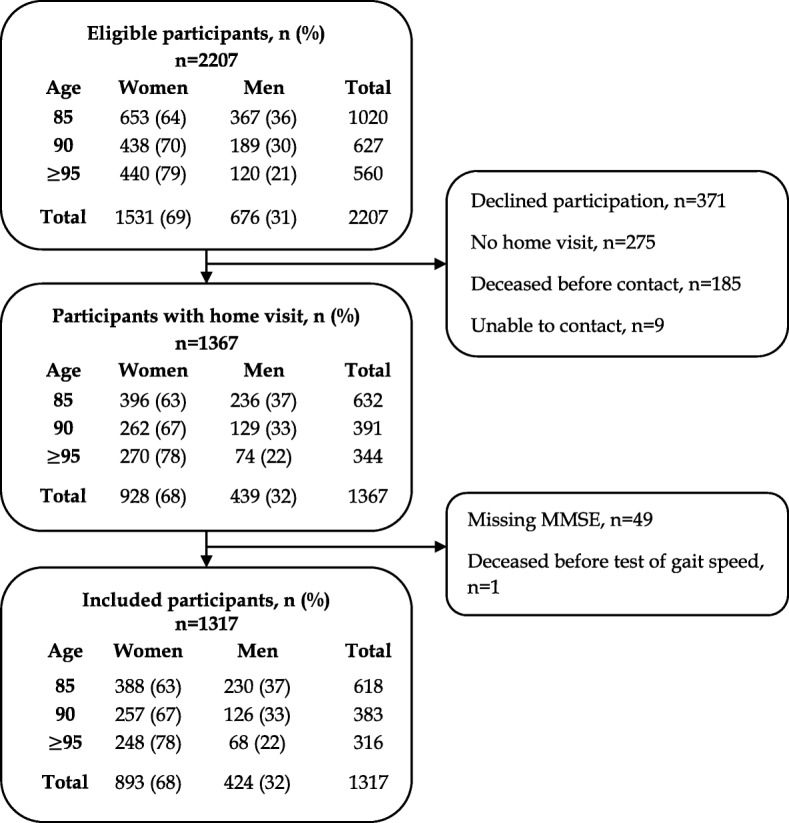
Flow chart of inclusion procedure. *MMSE = Mini-Mental State Examination*

This study included participants from the Umeå85+/Gerontological Regional Database (GERDA) study; a population-based cohort conducted by Umeå University, Sweden, Åbo Academy University/University of Vaasa, and Novia University of Applied Sciences, Finland. The purpose of the Umeå85+/GERDA-study is to increase the knowledge about health and living conditions among the oldest proportion of the population. The data collection has been 5-year recurrent in Västerbotten County, Sweden since year 2000. In Österbotten County, Finland, data has been collected in years 2005 and 2010. Eligible participants are inhabitants of selected urban and rural municipalities, who are chosen from national tax and population registers according to age. From a randomized starting point every other 85-, every 90-, and 95-year-old or older received a letter detailing the GERDA study and were thereafter contacted by telephone and offered participation. All included participants gave informed consent, with assent given by relatives in case of cognitive impairment. Trained assessors with medical knowledge (physicians, nurses, physical therapists) collected data by structured interviews and tests in the participants’ homes, a review of medical records, and interviews with relatives or care personnel when required. The present study included individuals that participated in the Mini-Mental State Examination (MMSE) in years 2000/2002, 2005/2007 and 2010/2012 (Fig. 1). Compared with participants in the study, amongst individuals that declined participation or had no MMSE score, a larger proportion were women (68% vs. 74% respectively, *p* = 0.008) but no differences were observed in age (*p* = 0.269). The earliest values of participants who took part in more than one data collection were used in analyses to reduce survival bias.

### Target variables

Self paced gait speed was measured in participant’s homes [18]. Participants were instructed to start from standstill behind one marking on the floor, and walk at usual pace past a second marking 2.4 m away. Time from when walking started to when the first foot passed the second marking was measured using a stopwatch. The test was performed twice, and mean gait speed calculated (meters/second, m/s). If only one measure was registered (*n* = 25) it was included in the analysis. The use of a walking aid was allowed and type recorded (cane, rollator, other). If the gait speed test could not be performed (*n* = 287) the cause was recorded and categorized as cognition, physical impairment, motivation, or other (e.g. pain, unknown).

Global cognitive function was assessed using the MMSE (0–30), with a higher score indicating better cognitive function. The MMSE is commonly used, and comprises eleven questions related to memory, attention, visuospatial function, and orientation to time and place. It is a valid and reliable screening tool for cognitive function [19, 20]. A minimal score was given on single items on the MMSE when participants refused to answer, or when prevented by vision, hearing, or motor impairments.

### Potential confounders

Sociodemographic data was recorded. Body weight and height was measured, and Body Mass Index (BMI, kg/m^2^) calculated. Systolic and diastolic blood pressure was measured using a calibrated manual sphygmomanometer and stethoscope, after 5 min rest in supine position. The 15-item Geriatric Depression Scale (GDS-15, 0–15) was used to measure depressive symptoms, with higher scores indicating greater depressive symptoms. When ≤5 answers were missing, a total score was imputed using the mean of answered questions multiplied by 15 [21]**.** Dependence in personal ADLs was measured using the Barthel ADL Index (0–20) with higher score indicating greater independence. Vision was rated as impaired when unable to read a word printed in 5-mm capital letters, with or without glasses. Hearing was rated as impaired when unable to hear a conversation held at usual speaking voice from a distance of 1 m, with or without a hearing aid. Information regarding past medical history and use of medication was collected in the interview, and by review of medical records. One experienced specialist in geriatric medicine reviewed all medical diagnoses in Sweden and Finland. Dementia diagnoses and depressive disorders were verified according to the Diagnostic and Statistical Manual of Mental Disorders, fourth edition, Text Revision [22] using available information from medical records, prescribed medication, and assessments including MMSE, GDS-15, Philadelphia Geriatric Center Morale Scale, Life Orientation Scale, Organic Brain Syndrome Scale, and hearing or vision impairment.

### Statistical analysis

The Chi-Square test or Student’s t-test, as appropriate, were used to analyze differences in target variables and potential confounders in the subgroups; between measured or missing gait speed values, and according to dementia.

The univariate and multivariate association between MMSE and gait speed (dependent variable) was investigated in linear regression analyses. From pre-selected variables related to either MMSE or gait speed, possible confounders were chosen based on a bivariate association (*p* ≤ 0.15) with both target variables (Additional file 1). Barthel ADL Index correlated with nursing home resident (r = − 0.61), dementia (r = − 0.57), and use of walking aids (r = − 0.52) and was excluded from multivariate analyses, since the latter two were variables of interest. Diastolic blood pressure was excluded in favor of systolic blood pressure (r = 0.52), since the less important risk factor for cardiovascular disease of the two. Both GDS-15 (r = 0.59), and use of antidepressants (r = 0.59) correlated with depressive disorders, and were removed. Depressive disorders were replaced by GDS-15 in a sensitivity analysis, and the results remained essentially the same. The remaining variables in the model were within reasonable limits regarding multicollinearity, distribution, and outliers.

Multiple imputation was chosen over single imputation techniques, since unlikely that all the missing gait speed values were missing completely at random (MCAR) [23]. Twenty imputed data sets were generated using a pre-defined strategy; predictors were selected from background characteristics that were, (i), target variables in the model, (ii), associated (r > 0.3) with target variables or, (iii), associated (r > 0.3) with causes for missing gait speed values [24]. Using known causes of missingness, i.e. physical impairment (*n* = 182), cognitive impairment (*n* = 24), motivation (*n* = 37), other (*n* = 27), or reason unknown (*n* = 17), two different restrictions were implemented using the same predictors based on the total sample. Imputed values for missingness due to physical impairments were limited to lowest observed value (0.08 m/s), since conceivably, if able to walk the pace would be slow. Whereas, for all other causes, with a greater uncertainty regarding prediction of pace, the whole range of observed gait speed values was allowed (0.08–1.5 m/s).

The univariate and multivariate association between MMSE and gait speed were further investigated in subgroups according to dementia diagnosis or use of walking aids, and the analyses repeated in each subgroup. Use of walking aid was dichotomized into without versus any type, of which 253 (79%) used a rollator. Differences in the association between MMSE and gait speed according to walking aid use were analyzed by adding an interaction term (walking aid use x MMSE) to the multivariate regression model. The strong association between MMSE and dementia precluded interaction analysis according to dementia diagnosis.

Analyses were performed using IBM SPSS Statistics for Windows version 23 software (IBM Corp., Armonk, NY). All statistical tests were two-tailed and *P* values < 0.05 were considered to be statistically significant.

## Results

Out of 2013 possible, 1317 participants (participation rate 65%) were included (Table 1) with mean age of 89.4 ± 4.6 years, mean MMSE score of 21.1 ± 7.8, and 68% were women. Gait speed was measured in 1030 (78%) participants with a mean gait speed of 0.45 ± 0.26 m/s, of which 321 (31%) used a walking aid.

**Table 1:** Characteristics of participants, total sample and according to ability to perform gait speed test

Characteristic	Total *n* = 1317	Measured GS *n* = 1030	Missing GS *n* = 287
Age, years (range)	89.4 ± 4.6 (84–103)	88.7 ± 4.3 (84–103)	91.6 ± 4.9** (84–103)
Age group, years, n (%)			
85	618 (46.9)	535 (51.9)	84 (29.2)
90	383 (29.1)	302 (29.3)	81 (28.1)
≥95	316 (24.0)	193 (18.7)	123 (42.3)
Women, n (%)	893 (67.8)	670 (65.0)	224 (77.8)**
Nursing home resident, n (%), *n = 1314*	462 (35.2)	266 (25.9)	196 (68.3)**
Lives alone, n (%), *n = 1311*	1014 (77.3)	770 (75.0)	244 (85.9)**
Education < 8 years, n (%), *n = 1278*	897 (70.2)	698 (68.9)	199 (75.1)*
Currently smoking, n (%), *n = 1308*	39 (3.0)	31 (3.0)	8 (2.8)
Diagnoses and medical conditions, n (%)			
Dementia disorder	464 (35.2)	256 (24.9)	208 (72.2)**
Parkinson’s disease	22 (1.7)	14 (1.4)	8 (2.8)
Depressive disorders	446 (33.9)	313 (30.4)	134 (46.5)**
Cerebrovascular disease	260 (19.7)	180 (17.5)	81 (28.1)**
Myocardial infarction previous year	33 (2.5)	25 (2.4)	8 (2.8)
Heart failure	397 (30.1)	276 (26.8)	122 (42.4)**
History of hip fracture	219 (16.6)	142 (13.8)	77 (26.7)**
Diabetes	221 (16.8)	171 (16.6)	50 (17.4)
Osteoarthritis	608 (46.2)	473 (45.9)	136 (47.2)
Malignancy previous 5 years	165 (12.5)	138 (13.4)	27 (9.4)
Routine prescription medications, n (%)			
Benzodiazepines	366 (27.8)	267 (25.9)	99 (34.4)*
Beta-blockers	500 (38.0)	418 (40.6)	82 (28.5)**
Antidepressants	239 (18.1)	150 (14.6)	90 (31.3)**
Diuretics	676 (51.3)	520 (50.5)	157 (54.5)
Analgesics	517 (39.3)	340 (33.0)	178 (61.8)**
Neuroleptics	149 (11.3)	77 (7.5)	73 (25.3)**
Number of prescribed medications	6.6 ± 4	6.3 ± 4.0	7.8 ± 3.7**
Assessments			
Systolic blood pressure, *n = 1268*	146.8 ± 23.3	149.6 ± 22.7	135.9 ± 22.3**
Diastolic blood pressure, *n = 1264*	74.4 ± 12.0	75.1 ± 12.0	71.6 ± 11.9**
Barthel ADL Index (0–20), *n = 1310*	16.5 ± 5.5	18.4 ± 2.8	9.4 ± 6.7**
Geriatric Depression Scale (0–15), *n = 1134*	3.6 ± 2.6	3.4 ± 2.6	4.7 ± 2.9**
Mini-Mental State Examination (0–30)	21.1 ± 7.8	23.4 ± 5.7	13.4 ± 9.1**
Vision impairment, n (%), *n = 1271*	203 (16.0)	119 (11.7)	84 (33.1)**
Hearing impairment, n (%), *n = 1302*	235 (18.0)	141 (13.8)	94 (32.8)**
Used walking aid in gait speed test, n (%), *n = 1024*	321 (31.3)	321 (31.4)	N/A
Gait Speed, m/s	0.45 ± 0.26 ^‡^	0.53 ± 0.22	0.18 ± 0.22 ^†^ **

Data presented as mean ± standard deviation (SD), unless stated otherwise. Geriatric Depression Scale: higher score indicate more depressive symptoms. Difference in means or proportions between group with measured vs. missing gait speed values, at:

**p < 0.001

*p < 0.05

^‡^Measured and imputed Gait Speed values

^†^Imputed Gait Speed values

When compared with participants that had measured gait speed, those missing a gait speed value were more likely to be older, female, and living in nursing homes (Table 1). Further, a larger proportion had depression, heart failure, cerebrovascular disease, or history of hip fracture, as well as more medications including analgesics and neuroleptics. In addition, participants with missing gait speed values had a lower cognitive function, and were more dependent in ADLs, and a larger proportion had dementia (72% vs. 25%, respectively). In the total sample, 464 (35%) had dementia (Table 2). Compared with participants without dementia, those with dementia had a higher proportion of depression, heart failure, history of hip fracture, medications, and they scored worse on most assessments (Table 2). In participants with dementia, those unable to perform the gait speed test due to physical reason (*n* = 140) had a mean MMSE score of 9.1 ± 7.3, while those able to perform the test (*n* = 256) had 16.1 ± 6.0.

**Table 2:** Characteristics of participants according to dementia disorder

Characteristic	No dementia *n* = 853	Dementia n = 464
Age, years (range)	88.4 ± 4.2 (84–103)	91.1 ± 4.9** (84–103)
Age group, years, n (%)		
85	472 (55.3)	146 (31.5)
90	243 (28.5)	140 (30.2)
≥95	138 (16.2)	178 (38.4)
Women, n (%)	548 (64.2)	345 (74.4)**
Nursing home resident, n (%), *n = 1314*	155 (18.2)	307 (66.2)**
Lives alone, n (%), *n = 1311*	632 (74.1)	382 (83.4)**
Education < 8 years, n (%), *n = 1278*	561 (66.3)	336 (77.8)**
Currently smoking, n (%), *n = 1308*	33 (3.9)	6 (1.3)*
Diagnoses and medical conditions, n (%)		
Parkinson’s disease	14 (1.6)	8 (1.7)
Depressive disorders	225 (26.4)	221 (47.6)**
Cerebrovascular disease	159 (18.6)	101 (21.8)
Myocardial infarction previous year	22 (2.6)	11 (2.4)
Heart failure	226 (26.5)	171 (36.9)**
History of hip fracture	111 (13.0)	108 (23.3)**
Diabetes	147 (17.2)	74 (15.9)
Osteoarthritis	414 (48.5)	194 (41.8)*
Malignancy previous 5 years	124 (14.5)	41 (8.8)*
Routine prescription medications, n (%)		
Benzodiazepines	215 (25.2)	151 (32.5)*
Beta-blockers	365 (42.8)	135 (29.1)**
Antidepressants	91 (10.7)	148 (31.9)**
Diuretics	431 (50.5)	245 (52.8)
Analgesics	260 (30.5)	257 (55.4)**
Neuroleptics	44 (5.2)	105 (22.6)**
Number of prescribed medications	6.2 ± 4.0	7.4 ± 3.8**
Assessments		
Systolic blood pressure, *n = 1268*	150.9 ± 22.6	138.8 ± 22.5**
Diastolic blood pressure, *n = 1264*	75.5 ± 11.8	72.3 ± 12.4**
Body Mass Index, *n = 1262*	25.9 ± 4.2	25.0 ± 4.7*
Barthel ADL Index (0–20), *n = 1310*	18.7 ± 2.6	12.2 ± 6.6**
Geriatric Depression Scale (0–15), *n = 1134*	3.3 ± 2.5	4.2 ± 3.0**
Mini-Mental State Examination (0–30)	25.4 ± 3.3	13.2 ± 7.4**
Vision impairment, n (%), *n = 1271*	89 (10.5)	114 (27.1)**
Hearing impairment, n (%), *n = 1302*	93 (11.0)	142 (31.3)**
Used walking aid in gait speed test, n (%), *n = 1024*	185 (24.0)	136 (53.5)**
Gait Speed, m/s	0.54 ± 0.24 ^‡^	0.30 ± 0.23 ^‡^ **
Missing Gait Speed values, n (%)	79 (9.3)	208 (44.8)

Data presented as mean ± standard deviation (SD), unless stated otherwise. Geriatric Depression Scale: higher score indicate more depressive symptoms. Difference in means or proportions between group with measured vs. missing gait speed values, at:

**p < 0.001

*p < 0.05

^‡^Measured and imputed Gait Speed values

The univariate and multivariate associations between MMSE and gait speed in the total sample, and in the subgroups according to dementia and use of walking aid are shown in Table 3. In the total sample, MMSE was associated with gait speed in the multivariate analysis, unstandardized beta (β) 0.006 m/s, 95% confidence intervals [CI] = 0.004, 0.008, *p* < 0.001; Table 3). When imputed values for gait speed were added, the association remained (β 0.011 m/s, 95%CI = 0.009, 0.013, p < 0.001).

**Table 3:** Association between Mini-Mental State Examination score and gait speed

	n	Univariate β (95% CI)	*p*-value	Multivariate β (95% CI)	*p*-value
Total sample					
GS measured	1030	0.015 (0.013, 0.017)	<0.001	0.006 (0.004, 0.008)	<0.001
GS measured+imputed	1317	0.018 (0.016, 0.020)	<0.001	0.011 (0.009, 0.013)	<0.001
Dementia					
GS measured	256	0.008 (0.005, 0.012)	<0.001	0.003 (0.000, 0.006)	0.058
GS measured+imputed	464	0.013 (0.010, 0.016)	<0.001	0.007 (0.002, 0.011)	0.002
No dementia					
GS measured	774	0.020 (0.015, 0.025)	<0.001	0.010 (0.006, 0.015)	<0.001
GS measured+imputed	853	0.024 (0.019, 0.029)	<0.001	0.015 (0.010, 0.020)	<0.001
No walking aid ^a^					
GS measured	703	0.012 (0.009, 0.016)	<0.001	0.010 (0.06, 0.014)	<0.001
Walking aid ^a^					
GS measured	321	0.007 (0.005, 0.009)	<0.001	0.005 (0.002, 0.008)	<0.001

From multivariate linear regression analyses adjusted for age, sex and baseline characteristics associated (p ≤ 0.15) with Gait Speed (GS) (measured + imputed) and Mini-Mental State Examination score: lives alone, education < 8 years, current smoker, depression, cerebrovascular disease, heart failure, history of hip fracture, malignancy previous 5 years, benzodiazepines, beta-blockers, analgesics, neuroleptics, number of prescribed medications, systolic blood pressure, vision impairment, hearing impairment and use of walking aid during gait speed test. In subgroup analyses with/without walking aids use of walking aids was omitted

^a^Participants who were unable to perform the GS test, and subsequently had a GS value imputed (n=287), had no reported walking aid and could therefore not be included in subgroup analyses of walking aid use

β unstandardized beta

In participants with dementia, the multivariate analyses indicated that, while MMSE was not significantly associated with gait speed in participants with measured gait speed (β 0.003 m/s, 95%CI = 0.000, 0.006, *p* = 0.058), when imputed values for gait speed were added, the association was significant (β 0.007 m/s, 95%CI = 0.002, 0.011, *p* = 0.002; Table 3). In participants without dementia, MMSE was found to be associated with gait speed and when imputed gait speed values were added the association remained (Table 3).

**Fig. 2:**
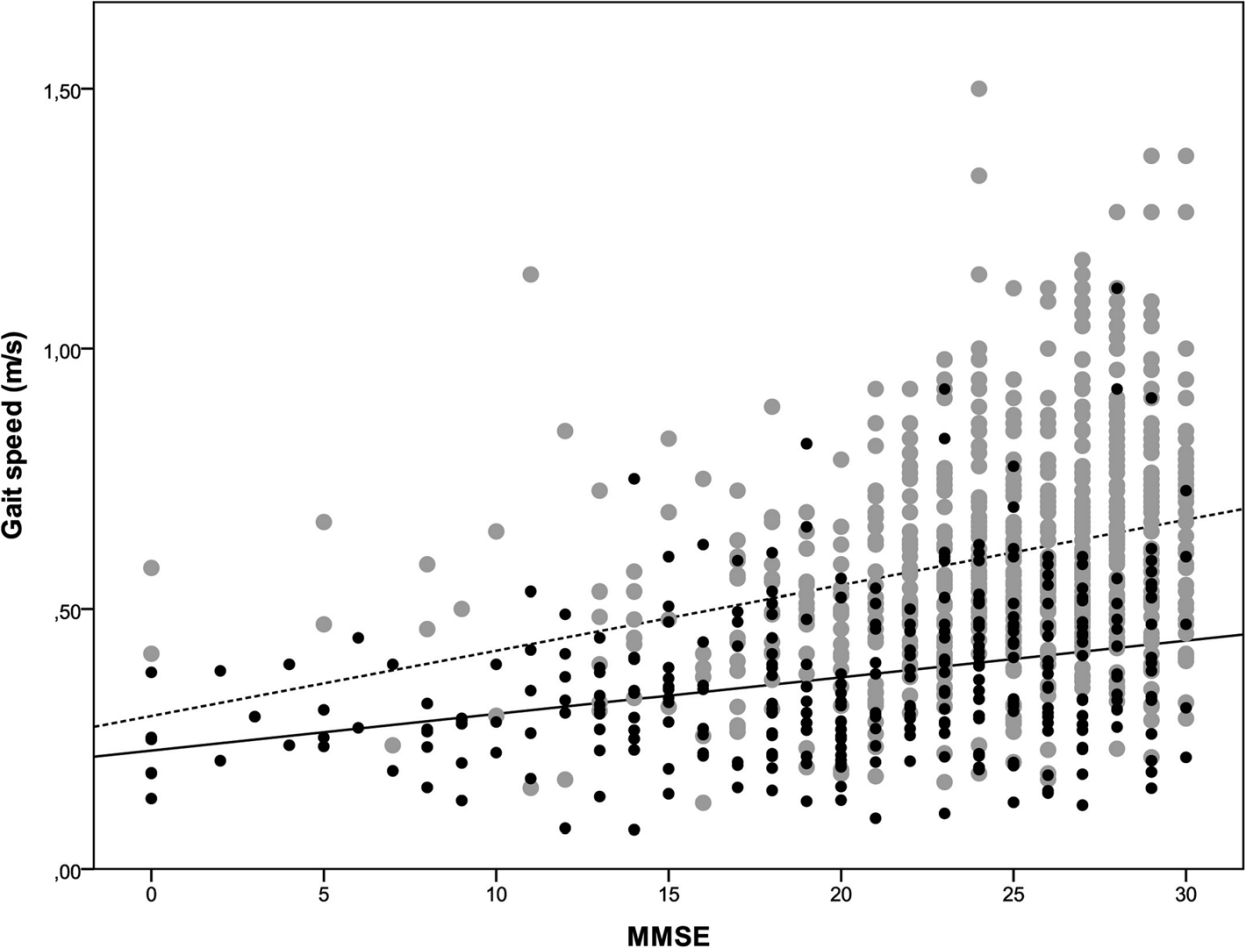
Univariate association between Mini-Mental State Examination (MMSE) score and Gait Speed (GS). *Gray dots (dotted line) represent participants not using walking aid during GS test, (n = 703, R*^*2*^ *= 0.073), while black dots (solid line) represent participants using walking aid during GS test, (n = 321, R*^*2*^ *= 0.098)*

In subgroup analyses according to walking aid use MMSE associated with gait speed irrespective of walking aid use (Table 3). In the interaction analysis with measured gait speed, the association differed between groups (β − 0.005 m/s, 95%CI = − 0.009, 0.000, *p* = 0.032) and was attenuated in participants using a walking aid; when imputed gait speed values were added the difference between groups remained (β − 0.019 m/s, 95%CI = − 0.024, − 0.013, *p* < 0.001). The univariate association between MMSE and gait speed according to walking aid use is shown in Fig. 2.

## Discussion

In this large representative study of very old people, which included people with dementia and people living in nursing homes, a positive association was found between global cognitive function and self paced gait speed when adjusted for potential confounders. When the association was analyzed in subgroups according to dementia diagnosis, no association between cognitive function and gait speed was observed in participants with dementia. However, an association was observed when participants with missing gait speed values, of who the majority had dementia, were added. In addition, the interaction analysis indicated that the association differed according to walking aid use, with a weaker association suggested in participants that used a walking aid compared with those that did not.

The results from this study are in line with a recent systematic review examining the relationship between mobility and cognition in healthy older adults, where an association between gait and global cognitive function was found [25]. In comparison with the studies reviewed in that study, participants in our sample were older, and included people in nursing homes, people with dementia, and people unable to perform the gait speed test. Older populations have been described as heterogeneous, with large variations in physical and cognitive function, number of diseases and disorders, and prescribed medications, which the participant characteristics in our study also supports. Despite the apparent heterogeneity, a positive association between cognitive function and gait speed was still found independent of many factors that could confound the association.

Mobility limitations become more prevalent with age, and have been reported to approach 90% in some nursing home settings [25, 26], which may subsequently impact ability to perform a gait speed test. One previous study of very old people showed that the ability to perform the gait speed test declined with age in women, with more than half of the female participants aged 95 years or older missing a gait speed value [27]. Studies investigating gait speed in older populations, both in clinical [28] and nursing home settings [17], have highlighted that most studies exclude persons that cannot walk. The same exclusion criterion is used by most studies investigating the association between gait and cognition [25], and may restrict the interpretation of results. In our study, participants with missing gait speed values constituted around one fifth of the sample, were older, had more prescribed medications, more diagnoses and medical conditions, and scored worse on most assessments, compared to participants with measured gait speed. Notably, the majority of participants missing a gait speed value had dementia and severe cognitive impairment.

In people with dementia, an association between cognitive function and gait speed was found in unadjusted analysis, which is in line with the age-adjusted analyses of a previous study in older people with dementia [29]. Potential confounding factors that could influence the association between cognitive function and gait speed include medical conditions and medications, which are prevalent in people with dementia [30] and for which adjustments may be important. In adjusted analyses, no association between gait speed and cognitive function was found until participants with missing gait speed values were accounted for. Likewise, a previous cross-sectional study of 161 participants with dementia in nursing homes found no association between global cognitive function and gait speed in comprehensively adjusted analyses [14]. However, the study only included participants able to perform a gait speed test. In the present study, although the sample of participants with dementia was comparatively large (*n* = 464) more than half had a missing gait speed value, and the results changed when imputed gait speed values were added. The observed change may be due to participants with dementia and missing gait speed values having low MMSE scores. Ways to handle missing values are still debated. In our study, we used a pre-planned strategy, and included known causes for missingness according to recommendations [24]. The result suggests that excluding people with dementia that are unable to perform gait speed tests increases the risk of selection bias, and the excluded number should at the least be reported to aid interpretation.

In participants that used a walking aid the association between cognitive function and gait speed attenuated. Subsequently it appears that using a walking aid when walking straight ahead may decrease cognitive processing burden, possibly by compensating for poor balance, muscle strength, or lower limb motor control, or alleviating pain. The result is in line with a study of the association between backwards walking speed and cognitive function in older people with dementia living in nursing homes [14]. In addition, in a small study of healthy adults walking aid use reduced reaction time whilst beam-walking, indicating less cognitive load [31]. Conversely, more complex walking, for example turning, which requires greater maneuvering of the walking aid may increase cognitive load [15]. The impact of walking aid use on cognitive load needs further investigation, but in light of our result physical exercise may be indicated to augment gait, thus alleviate cognitive load and increase cognitive resources available for complex walking and to avoid falls.

The representativeness is a strength of this study of very old people, which included individuals with dementia and nursing home residents. Further, we included people that could not perform a gait speed test, which is a frequent exclusion criterion. Multiple imputation models were used to estimate missing values, which by adding uncertainty makes results more conservative, thus reducing the risk of type 1 errors [23]. However, this study is not without limitations. The comparatively short distance of 2.4 m were chosen due to concerns about limited space in participants’ homes, and since the acceleration phase constitutes a relatively large proportion of the measured distance, may have influenced the results. The MMSE was used to measure global cognitive function and covers some aspects of cognitive function but not all, e.g. executive function, which is associated with gait [7]. Further, the MMSE score may be influenced by factors besides cognitive function e.g. hearing, vision and motor deficits, which are prevalent in very old people. This can be a limitation when testing cognitive function with screening tests among very old people, and may affect the estimates. Low participation rate can be a problem in observational studies, and was 65% in our study. A higher proportion of women declined participation, or had no MMSE score, which may limit generalizability of results. Furthermore, inferences are limited to the age groups 85-, 90-, and 95 years or older. The cross-sectional design precluded inferences regarding causality, which requires randomized controlled designs. All walking aids were grouped together and prevented analyses according to types of walking aid.

## Conclusion

Global cognitive function appears to be independently associated with gait speed in very old people. However, in people with dementia selection bias was indicated since unless those with missing gait speed values were accounted for by imputation no association with cognition was observed. When walking straight ahead the use of a walking aid seemed to attenuate cognitive load, which may not apply in daily activities, and requires further investigation.

## Supplementary information

**Additional file 1:** Univariate association of characteristics with self paced gait speed and Mini Mental State Examination (MMSE).

## Abbreviations

ADL: Activities of daily living
BMI: Body Mass Index, bodyweight in kilograms/height in meters squared
GDS-15: The 15-item geriatric depression scale
GERDA: Gerontological regional database
M/s: Meters per second
MMSE: Mini-Mental State Examination
