# Supplementary material for: The association between cognition and gait in a representative sample of very old people – the influence of dementia and walking aid use
Source: BMC Geriatr. 2020 Jan 31;20:34. doi: 10.1186/s12877-020-1433-3 (PMC6995040; doi:10.1186/s12877-020-1433-3)
Supplement: Supplementary file 1 — Additional file 1: Univariate association of characteristics with self paced gait speed and Mini Mental State Examination (MMSE). [file 12877_2020_1433_MOESM1_ESM.docx]

**Additional file 1** Univariate association of characteristics with self paced gait speed and Mini Mental State Examination (MMSE)

| **Characteristic** | Total  n=1317 | Gait speed  β (95% CI) | p-value | MMSE  β (95% CI) | p-value |
| --- | --- | --- | --- | --- | --- |
| **Age, mean ± SD** | 89.4 ± 4.6 | -0.022 (-0.025, -0.019) | **<0.001** | -0.565 (-0.650, -0.479) | **<0.001** |
| **Age group, n (%)** |  | -0.127 (-0.143, -0.110) | **<0.001** | -3.208 (-3.696, -2.721) | **<0.001** |
| 85 | 618 (46.9) |  |  |  |  |
| 90 | 383 (29.1) |  |  |  |  |
| ≥ 95 | 316 (24) |  |  |  |  |
| **Women, n (%)** | 893 (67.8) | -0.120 (-0.151, -0.089) | **<0.001** | -2.571 (-3.458, -1.685) | **<0.001** |
| **Nursing home resident (%),** *n=1314* | 462 (35.2) | -0.271 (-0.298, -0.244) | **<0.001** | -9.321 (-10.040, -8.601) | **<0.001** |
| **Lives alone (%),** *n=1311* | 1014 (77.3) | -0.138 (-0.172, -0.103) | **<0.001** | -3.210 (-4.194, -2.225) | **<0.001** |
| **Education < 8 years, n (%),** *n=1278* | 897 (70.2) | -0.066 (-0.098, -0.033) | **<0.001** | -3.215 (-4.094, -2.335) | **<0.001** |
| **Currently smoking, n (%),** *n=1308* | 39 (3.0) | 0.104 (0.016, 0.191) | **0.020** | 2.912 (0.461, 5.363) | **0.020** |
| Diagnoses and medical conditions, n (%) |  |  |  |  |  |
| **Dementia** | 464 (35.2) | -0.244 (-0.273, -0.215) | **<0.001** | -12.160 (-12.742, -11.578) | **<0.001** |
| Parkinson’s disease | 22 (1.7) | -0.149 (-0.262, -.037) | 0.009 | -0.051 (-3.323, 3.221) | 0.976 |
| **Depression** | 446 (33.9) | -0.129 (-0.160, -0.098) | **<0.001** | -3.287 (-4.156, -2.419) | **<0.001** |
| **Cerebrovascular disease** | 260 (19.7) | -0.102 (-0.138, -0.066) | **<0.001** | -1.260 (2.311, -.208) | **0.019** |
| Myocardial infarction previous year | 33 (2.5) | -0.038 (-0.130, 0.054) | 0.421 | 1.083 (-1.600, 3.766) | 0.429 |
| **Heart failure** | 397 (30.1) | -0.122 (-0.153, -0.091) | **<0.001** | -1.702 (-2.611, -.793) | **<0.001** |
| **History of hip fracture** | 219 (16.6) | -0.141 (-0.179, -0.102) | **<0.001** | -3.496 (-4.604, -2.384) | **<0.001** |
| Diabetes | 221 (16.8) | -0.015 (-0.054, 0.024) | 0.448 | 0.190 (-0.933, 1.312) | 0.741 |
| Osteoarthritis | 608 (46.2) | -0.011 (-0.040, 0.019) | 0.477 | 0.629 (-0.212, 1.469) | 0.143 |
| **Malignancy previous 5 y** | 165 (12.5) | 0.048 (0.005, 0.092) | **0.030** | 2.475 (1.215, 3.735) | **<0.001** |
| Routine prescription medication, n (%) |  |  |  |  |  |
| **Benzodiazepines** | 366 (27.8) | -0.082 (-0.114, -0.050) | **<0.001** | -1.498 (-2.431, -0.566) | **0.002** |
| **Beta-blockers** | 500 (38.0) | 0.050 (0.020, 0.080) | **0.001** | 2.586 (1.733, 3.439) | **<0.001** |
| **Antidepressants** | 239 (18.1) | -0.157 (-0.195, -0.120) | **<0.001** | -5.050 (-6.103, -3.996) | **<0.001** |
| Diuretics | 676 (51.3) | -0.074 (-0.103, -0.045) | <0.001 | -0.060 (-0.899, 0.779) | 0.889 |
| **Analgesics** | 517 (39.3) | -0.180 (-0.209, -0.151) | **<0.001** | -4.622 (-5.444, -3.800) | **<0.001** |
| **Neuroleptics** | 149 (11.3) | -0.186 (-0.232, -0.141) | **<0.001** | -7.335 (-8.598, -6.072) | **<0.001** |
| **Number of prescribed drugs** | 6.6 ± 4 | -0.018 (-0.022, -0.014) | **<0.001** | -0.305 (-0.410, -0.201) | **<0.001** |
| Assesments |  |  |  |  |  |
| Body mass index, *n=1262* | 25.6 ± 4.4 | 0.002 (-0.001, 0.006) | 0.206 | 0.244 (0.153, 0.335) | <0.001 |
| **Systolic blood pressure**, *n=1268* | 146.8 ± 23.3 | 0.003 (0.002, 0.003) | **<0.001** | 0.086 (0.069, 0.104) | **<0.001** |
| **Diastolic blood pressure**, *n=1264* | 74.4 ± 12.0 | 0.003 (0.002, 0.005) | **<0.001** | 0.097 (0.064, 0.131) | **<0.001** |
| **Barthel ADL Index (0-20)**, *n=1310* | 16.5 ± 5.5 | 0.031 (0.029, 0.033) | **<0.001** | 1.045 (0.993, 1.097) | **<0.001** |
| **Geriatric Depression Scale** (0-15), *n=1134* | 3.6 ± 2.6 | -0.027 (-0.032, -0.021) | **<0.001** | -0.460 (-0.571, -0.349) | **<0.001** |
| Mini-Mental State Examination (0-30) | 21.1 ± 7.8 | 0.018 (0.016, 0.020) | <0.001 | ---- |  |
| **Vision impairment, n (%),** *n=1271* | 203 (16) | -0.158 (-0.198, -0.118) | **<0.001** | -6.554 (-7.545, -5.562) | **<0.001** |
| **Hearing impairment, n (%),** *n=1302* | 235 (18) | -0.147 (-0.185, -0.110) | **<0.001** | -6.622 (-7.630, -5.614) | **<0.001** |
| **Used walking aid in GS test, n (%),** *n=1024* | 321 (31.3) | -0.276 (-0.307, -0.245) | **<0.001** | -4.286 (-5.003,-3.570) | **<0.001** |
| Gait speed m/s | 0.45 ± 0.26^‡^ | ---- |  | 15.638 (14.184, 17.093) | <0.001 |

Data presented as mean ± standard deviation unless stated otherwise. Bold variabels associated (p < 0.15) with gait speed and Mini Mental State Examination score. Geriatric Depression Scale: higher score indicate more depressive symptoms.

‡ Measured and imputed GS values.

β unstandardized beta.
